# Impact of cavity shave margins in patients with ductal carcinoma *in situ* undergoing conserving breast surgery

**DOI:** 10.3389/fonc.2024.1403069

**Published:** 2024-05-16

**Authors:** Gianluca Vanni, Marco Pellicciaro, Marco Materazzo, Valentina Marsella, Valeria Usai, Annalisa Noce, Oreste Claudio Buonomo

**Affiliations:** ^1^ Breast Unit Policlinico Tor Vergata, Department of Surgical Science, Tor Vergata University, Rome, Italy; ^2^ PhD Program in Applied Medical-Surgical Sciences, Department of Surgical Science, Tor Vergata University, Rome, Italy; ^3^ UOSD Nephrology and Dialysis, Policlinico Tor Vergata, Rome, Italy; ^4^ Department of Systems Medicine, University of Rome Tor Vergata, Rome, Italy; ^5^ University of Basilicata, Potenza, Italy; ^6^ Breast Unit, Policlinico Tor Vergata, Rome, Italy

**Keywords:** ductal carcinoma *in situ*, cavity shave, positive margins, reduce re-excision, breast cancer

## Abstract

**Aim:**

The main challenge during breast-conserving surgery (BCS) is to obtain clear margins, especially in patients with ductal carcinoma *in situ* (DCIS) due to the absence of well-defined nodules. Many surgical approaches have been used in an attempt to reduce the positive margin rate. The aim of this retrospective study is to compare the cavity shave margin technique with standard surgery and the intraoperative evaluation of surgical margins.

**Methods:**

This is a single-center retrospective study analyzing margin status, need for re-excision, and surgical time in a cohort of 227 patients who underwent surgery from September 2016 to September 2022.

**Results:**

In patients subjected to cavity shaving, we reported a significant reduction in positive margins of 17.1% versus 28.7% (*p*-value = 0.042). Also, a difference in terms of surgical re-excision was reported as *p*-value = 0.039 (12.4% versus 23.8%, respectively, for the cavity shave and control group). In the multivariate analysis, intraoperative evaluation of the margins was a risk factor for margins re-excision (Wald = 4.315, *p* = 0.038, OR: 2.331 [95% CI: 1.049–5.180]). Surgical time was lower in patients subjected to cavity shaves (*p* = 0.024), and the relative mean time was 68.4 min ± 37.1 min in the cavity shave group versus 93.9 min ± 40.6 min in the control group.

**Conclusion:**

The cavity shave margin technique in conserving breast surgery results in a reduction in positive margin rate, surgical re-excision, and operative time.

## 1 Introduction

Ductal carcinoma *in situ* (DCIS) is a malignant epithelial cell proliferation confined to the myoepithelial cell’s basement membrane (1). During the past 20 years, the incidence of such disease has increased by approximately 25% of all new breast cancer diagnoses (1). The surgical treatment for the invasive carcinoma consists of either breast-conserving surgery (BCS) or mastectomy with an equivalent overall and recurrence-free survival (2–4). The main challenge for the surgeons during conserving breast surgery is to obtain clear or negative margins while saving normal tissue in order to achieve good aesthetic results (5). It has been proven that negative margins reduce future local recurrence. According to the 2016 guidelines for breast-conserving surgery in DCIS, published by the Society of Surgical Oncology and the Society of Radiation Oncology, oncological safety is reached when the distance between the lesion and the resection margin is ≥ 2 mm (6). However, clinical judgment takes precedence when margin safety is not obtained during the first surgery.

In several clinical trials, resection of the cavity shave margin was found to reduce positive margin rates by at least 50% in breast-conserving surgery for invasive cancer (7–9). To our knowledge, one randomized controlled trial evaluating the impact of cavity shave margins was reported in the literature, demonstrating an advantage in terms of negative margin for this technique (10).

DCIS are known to have a different growth pattern when compared to invasive cancer. Lesions are rarely nodular, but they have the tendency to grow in a more discontinuous or skip-like fashion, especially in low-grade DCIS (11). This feature could indicate a need for wider margins.

Intraoperative evaluation of resection margins is now standardized in breast-conserving surgery in order to achieve negative margins. However, due to the absence of clear nodules, intraoperative evaluation of resection margins presented several limitations for DCIS and a higher rate of resulting “false positives” (10).

The aim of this retrospective study was to evaluate the potential benefits of cavity shaves for the management of resection margins in DCIS treated with breast-conserving surgery.

## 2 Materials and methods

A single-center retrospective study including all patients with a diagnosis of DCIS who underwent BCS from September 2016 to September 2022 was evaluated by the Breast Unit of the Policlinico Tor Vergata, Rome. The study was approved by the local ethics committee (approval number 12/24). All data were retrieved from clinical notes and surgical and pathological reports.

Preoperative diagnosis was achieved by fine needle aspiration, microbiopsy, vacuum-assisted biopsy, or vacuum-assisted excision and replated from preoperative histological examination results.

Breast surgery was divided into breast-conserving surgery or mastectomy. Breast-conserving surgeries included all the procedures with partial gland removal. When possible, lumpectomy was the main procedure performed with oncoplastic principles. Oncoplastic level II surgeries were excluded from the study given the large resection volume and the technically longer surgical time.

Mastectomy is considered when a complete asportation of the gland is performed, including a skin or nipple-sparing mastectomy. Patients subjected to mastectomy were also excluded.

All axillary procedures for lymph node staging were evaluated in the study. Axillary surgery was performed in all patients with a high risk of lymph node involvement, such as high-grade CDIS, and clinical or radiological suspicion of axillary disease.

Removal of sentinel lymph with or without complementary nodes was classified as sentinel lymph node biopsy (SNLB); otherwise, it was considered an axillary lymph node dissection (ALND). Data regarding surgical incision and skin resection were collected from clinical notes. In our practice, we follow the breast cancer national guidelines (11). Therefore, any patient with a clinical or radiological suspicion of invasive cancer underwent axillary surgery in the first place. In one other ongoing study, we found that nodular lesions have a higher risk of upstaging. For this reason, we raise a discussion on the choice to perform SLNB in the first surgery or to delay it to a second surgery in case of invasion or microinvasion at the final histopathological exam, always in accordance with the patient’s preference.

The cohort was divided into two groups based on whether the intraoperative evaluation of margins was performed or not according to the surgical report, control, and cavity shave groups, respectively.

The number and site of margin widening after intraoperative evaluation were reported. The cavity shave group (CS group) includes all patients in whom the surgeon performed an additional circumferential resection of the tissue within the excision cavity, widening all margins with no need for an intraoperative evaluation. Cases of intraoperative specimen radiography were reported from surgical reports and analyzed.

Histopathological characteristics of the tumor were retrieved from the final pathological examination report, including nuclear grade and breast cancer prognostic and predictive factors such as estrogen receptor (ER) and progesterone receptor (PR), as indicated by the recommendations of the 2018 ASCO/CAP. Tumor dimension refers to the maximum diameter expressed in millimeters. Surgical margins were defined as the distance between tumoral cells and resection margins expressed in millimeters; all margins of > 2 mm were considered negative. A second surgery for positive margins performed within 120 days was considered a re-operation.

Surgical time, defined as the time in the operating room, was collected from operative records and reported in minutes.

### 2.1 Statistical analysis

Data were recorded into an EXCEL database (Microsoft, Redmond, WA, USA). Categorical variables were reported as the mean and standard deviation. The Mann–Whitney *U* test was used to compare two different groups. Continuous variables presented as numbers and percentages were analyzed using the Student’s *t*-test. The Chi-squared test (or Fisher’s exact test, depending on group size) is used to analyze categorical dichotomous variables. For no-dichotomous variables, the Monte Carlo test was adopted. All variables with a *p*-value of < 0.05 were considered statistically significant. Multivariate logistic regression analysis was used to assess the effect of the type of margin resection, independent of potential confounders. Statistical analysis was performed using SPSS statistical package version 23.0 (SPSS Inc., Chicago, IL, USA).

## 3 Results

**Figure 1:**
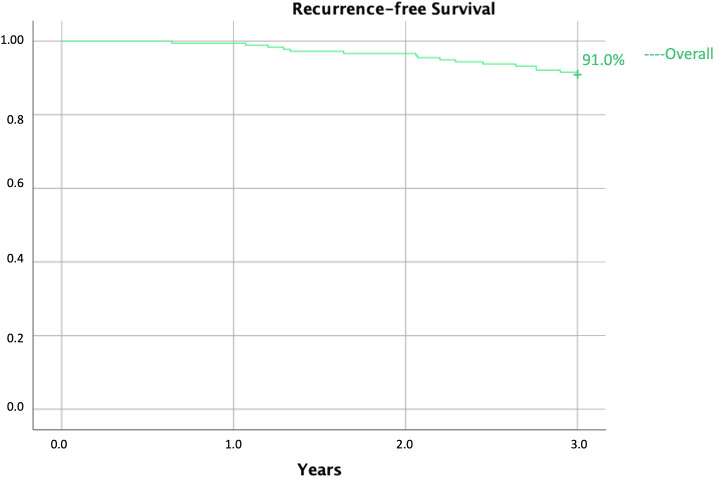
Overall 3-year recurrence-free survival.

From September 2016 to September 2022, 268 patients with a diagnosis of DCIS were evaluated at the Breast Unit of the Policlinico Tor Vergata, Rome. A total of 41 (15.3%) patients underwent a mastectomy and were excluded from the analysis. The 227 (84.7%) patients who underwent CBC were considered in this retrospective study. The mean age was 61.3 years ± 13.6 years, and the BMI was 24.4 ± 5.0. The mean follow-up was 4.1 years ± 1.9 years. In 53 (23.8%) patients, resection margin was < 2 mm, and 42 (18.5%) patients underwent re-operation for positive margins. At three years of follow-up, 16 (7.1%) patients presented homolateral recurrence (**Figure 1**). Out of these 16 patients, nine (56.2%) were diagnosed with DCIS, while seven (43.8%) were diagnosed with an invasive disease.

A total of 105 (46.2%) patients underwent cavity shaving (CS group), while 122 (53.8%) patients received intraoperative evaluation of margins and were considered the control group (C group). Age was comparable between the two groups 60.7 years ± 11.1 years versus 63.2 years ± 13.8 years (*p*-value = 0.789). In the CS group, BMI was 26.3 kg/m^2^ ± 5.5 kg/m^2^ versus 24.9 kg/m^2^ ± 4.7 kg/m^2^ (*p*-value = 0.079). The mean follow-up was 4.3 years ± 2.1 years in the CS group versus 4.0 years ± 1.8 years, *p*-value = 0.168.

The number of multifocal lesions was comparable in the CS group 6 (6.5%) versus 17 (13.9%) in the C group with a relative *p*-value of 0.079. All data regarding preoperative features are resumed in **Table 1**.

**Table 1:** Tumor preoperative characteristics and intraoperative findings between groups.

	CS group (*n* = 105)	C group (*n* = 122)	*p*-value
***Multifocality***	6 (6.5%)	14 (11.4%)	0.079
***Multicentricity***	0	1 (0.8%)	0.380
***Wire guide localization***	65 (69.1%)	95 (77.8%)	0.156
***Type of incision***			0.114
***Radial***	34 (30.3%)	29 (23.7%)	
***Periareolar***	30 (28.6%)	32 (26.2%)	
***Paraareolar***	8 (7.6%)	16 (13.1%)	
*Lesions quadrant*			
***Upper outer quadrant UOQ***	38 (36.2%)	60 (49.2%)	0.203
***UOQ-LOQ***	8 (7.6%)	11 (9.1%)	
***Upper inner quadrant UIQ***	9 (8.6%)	8 (7.1%)	
***LOQ-LIQ***	4 (3.8%)	15 (12.3%)	
***Lower outer quadrant LOQ***	9 (8.6%)	6 (4.9%)	
***Central portion***	2 (1.9%)	0 (0)	
***UOQ-UIQ***	12 (11.4%)	7 (5.7%)	
***Lower inner quadrant LIQ***	2 (1.91%)	2 (1.7%)	
***Specimen radiographs***	57(54.3%)	64 (52.5%)	0.269
***Removal of skin***	16 (17.9%)	35 (28.6%)	0.088
***Intraoperative evaluation SNLB***	8 (7.6%)	38 (31.1%)	**0.001**
***Upsgtaging***	11(10.1%)	17 (13.9%)	0.544
*Axillary surgery*			
***SNLB***	40 (38.1%)	49 (40.2%)	0.171
***ALND***	2 (1.9%)	5 (4.1%)	
***Omission***	63 (60.0%)	68(55.7%)	

SNLB, sentinel lymph node biopsy; ALND, axillary lymph node dissection.

The tumor dimension was comparable between groups: 10.5 mm ± 7.8 mm in the CS group vs 9.7 mm ± 6.4 mm in the C group (*p*-value = 0.439).

The type of surgical incision adopted by the surgeon for the BCS did not show any statistically significant difference between the two groups and a relative *p*-value of 0.114 (**Table 1**). The site of cancer according to the breast quadrant did not show any statistically significant difference *p* = 0.203. Distributions of lesions according to breast quadrant are displayed in **Table 1**. Skin removal during BCS did not show any statistical significance, and the relative *p*-value was 0.088, with an incidence rate of 17.9% (*n* = 16) in the CS group versus 28.6% (*n* = 35) in the C group. A total of 29 (27.6%) cases presented microcalcifications at preoperative mammography in the CS group versus 38 (31.3%) in the C group, and the relative *p*-value was 0.661. Patients who required wire-guided lesion localization before surgery were 65 (69.1%) in the CS group versus 95 (77.8%) in the C group, and the *p*-value was 0.156. In the CS group, 57 (54.3%) cases needed intraoperative specimen radiography versus 64 (52.5%) in the C group and a relative *p*-value of 0.269.

Intraoperative evaluation, with a frozen section, of sentinel lymph nodes was performed in eight (7.6%) patients in the CS group and in 38 (31.1%) in the control group, showing a significant statistical difference (*p*-value = 0.001). Axillary lymph node dissection was performed in two (1.9%) cases in the CS group versus five (4.1%) (*p*-value = 0.455). Four patients presented with an invasive disease with lymph node involvement; one of them underwent lymph node dissection, which had a negative final histopathological exam result. Two patients presented clinically suspicious lymph nodes, both positive at the final histopathological exam result; both of these latter patients were submitted to mastectomy to widen their margins; most probably, the invasive component was missed during the preoperative biopsy examination.

The CS group saw omission of sentinel biopsy in 63 cases (60.0%), compared to 68 cases (55.7%) in the control group. The difference was not statistically significant (*p*-value = 0.590). Similarly, no statistically significant difference was found between the two groups when comparing axillary procedures using the Monte Carlo test (*p*-value = 0.171) (**Table 1**).

In the CS group, 17.1% (*n* = 18) of resection margins were < 2 mm, therefore considered positive, compared to the 28.7% (*n* = 35) in the C group (*p*-value = 0.042).

The mean resection distance was 6.9 mm ± 0.5 mm in the CS group and 4.7 mm ± 0.4 mm in the C group, with a relative *p*-value of 0.001 (**Table 2**).

**Table 2:** Evaluation of margins between groups.

	*CS group (n = 105)*	*C group (*n *= 122)*	*p-value*
*Resection margin distance*			
***Deep margin (mm)***	9.3 ± 2.4	8.4 ± 3.5	**0.045**
***Superficial margin (mm)***	8.7 ± 3.1	7.6 ± 4.1	**0.041**
***Lateral margin (mm)***	9.3 ± 2.2	8.1 ± 3.7	**0.006**
***Medial margin (mm)***	8.8 ± 2.9	9.1 ± 2.8	0.469
***Upper margin (mm)***	9.8 ± 1.1	8.5 ± 3.4	**0.001**
***Lower margin (mm)***	9.4 ± 2.1	9.5 ± 2.1	0.744
*Closer margin*			
***Negative***	87 (82.8%)	87 (71.3%)	**0.001**
***Deep margin***	2 (1.9%)	2(1.6%)	
***Superficial margin***	6 (5.7%)	12 (9.8%)	
***Lateral margin***	2 (1.9%)	12 (9.8%)	
***Medial margin***	6 (5.7%)	0(0)	
***Upper margin***	0 (0)	8 (6.5%)	
***Lower margin***	2 (1.9%)	1 (0.8%)	
***Multiple positive margins***	4 (3.8%)	18 (15.7%)	**0.006**

Looking at the need for re-excision due to positive margins, 12.4% (*n* = 13) of the patients in the CS group needed a second surgery, compared to 23.8% (*n* = 29) in the C group, with a statistically significant *p*-value of 0.039.

**Figure 2:**
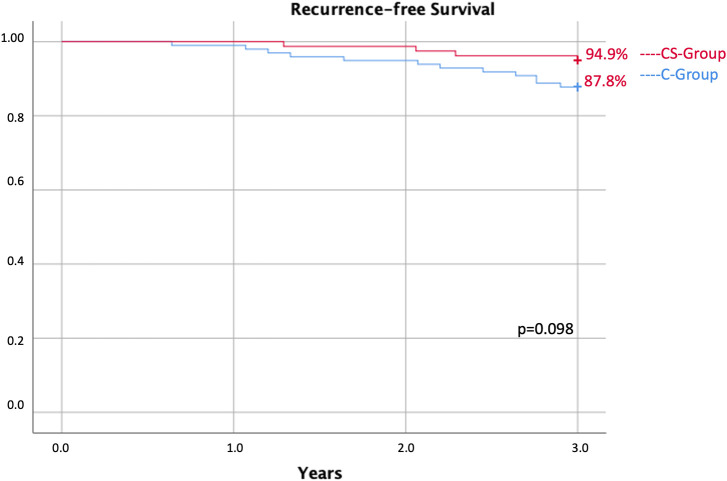
Recurrence-free survival of 3 years between the CS group and the C group.

The recurrence rate at 3-year follow-up was 5.1% in the CS group versus 12.2% in the C group; disease-free survival is shown in the Kaplan–Meier curve; and the relative log range was 0.098 (**Figure 2**).

The operative time for the different techniques was 71.2 min ± 37.6 min in the CS group, while in the C group it was 101.6 min ± 42.9 min, and the relative *p*-value was 0.002. Excluding patients subjected to intraoperative evaluation of the sentinel lymph node, the operative time was 68.4 min ± 37.1 min in the CS group versus 93.9 ± 40.6, *p*-value = 0.024.

In the univariate logistic regression analysis of the multifocal lesions, cancer grade and technique adopted for cavity or intraoperative evaluation of resection margins presented a *p*-value inferior to 0.010, and they were considered predictive factors of resection margins re-excision (**Table 3**). A multivariate logistic regression was performed to evaluate the effect of multifocal lesions, cancer grade, and intraoperative evaluation of lesions on the re-excision risk. According to the multivariate analysis, only intraoperative evaluation of the lesion was a risk factor for resection margin re-excision (Wald = 4.315, *p* = 0.038, OR: 2.331 [95% CI: 1.049–5.180]).

**Table 3:** Multivariate logistic regression for re-excision of margins.

Univariate			
Variables	OR	95% CI	*p*-value
**Multifocality**	0.503	0.245–1.032	0.061
**Tumor grade**	0.355	0.211–1.403	0.048
**Intraoperative evaluation of margins**	2.183	1.068–4463	0.032
Multivariate			
**Multifocality**	0.648	0.201–2.089	0.467
**Tumor grade**	0.681	0.222–1.521	0.067
**Intraoperative evaluation of margins**	2.331	1.049–5.180	0.038

OR, odds ratio; CI, confidence interval.

## 4 Discussion

The evaluation of patients submitted to BCS with a diagnosis of pure DCIS carried out in this retrospective study revealed that the cavity shave margin technique lowered the rate of positive margins. In our monocentric experience, 17.1% of patients in the CS group had a resection margin of < 2 mm and were therefore considered positive, compared with 28.7% in the C group. Moreover, we found that removing an extra layer of tissue, as in the cavity shave technique, lowered the rate of surgical re-excision by 12.4% versus 23.8% in the C group. Not taking the cavity shave margin resulted in an almost twofold increase in the need for surgical re-excision of the margins (OR: 2.331 [95% CI: 1.049–5.180]; *p*-value = 0.038), regardless of the type of lesions and DCIS grade. This outcome is similar to previous multicenter randomized controlled trials (10). In the same study, authors found a correlation between positive margins and lesion size (10). In our analysis, size is correlated with positive margins; however, this is outside the scope of the study. Moreover, in our study, the size is not a predictive factor of positive margins, and the technique adopted for the management of surgical margins is not correlated with lesion size. This difference could be explained by the improvement in diagnostic techniques such as magnetic resonance and contrast-enhanced mammography that nowadays allow a better preoperative evaluation of the lesion extension and therefore the real need for mastectomies.

Current literature shows how a preoperative evaluation of the real tumor extension with contrast-enhanced mammography can be detrimental to the surgical choice and therefore to obtaining both optimal oncological and esthetical results. A second-level exam such as CEM or MRI allows us to understand the extent of the needed resection prior to surgery. This most likely will ensure healthy tissue sparing for a better reconstruction of the left tissue without compromising the oncological outcome (12, 13).

DCIS is known to have a different pattern of growth, usually with a skip-like distribution; nodular lesions are less common, especially in low nuclear grade, as reported in a previous study performed by Faverly et al. (14). Merrill et al. reported that the majority of DCIS presents a multifocal distribution with a gap width of < 5 mm (15). Obtaining clear margins in patients with DCIS might therefore be problematic. We believe that routine cavity shaving could help the physician obtain a negative margin. Our previous retrospective analysis, comparing cavity shaving and intraoperative evaluation of resection margin by the pathologist in invasive cancer, highlighted a significant reduction of positive margins; however, there was no statistically significant difference in margins after re-excision (16). This dissimilarity between *in situ* lesions and invasive breast cancer could be justified by the different growth patterns between the lesions (16). Furthermore, the absence of tactile feedback from the nodule in DCIS lesions can make it more difficult to obtain a disease-free surgical margin, and especially in these patients, the cavity shaving technique could reduce the risk of positive margins and the need for surgical re-excision.

Many other techniques have also been used to reduce the positive margin rate in CBS. Racz et al., as in the control group of our study, analyzed 688 patients with a diagnosis of DCIS subjected to BCS and intraoperative evaluation of frozen sections of margins (17). They reported that about 63% of DCIS patients presented close or positive margins (17). Although our study also revealed an increased incidence of positive margins in patients subjected to the intraoperative frozen section of margin compared to cavity shave, the rate was lower than the above-cited study. Intraoperative analysis of resection margins has shown good results in terms of positive margin rate reduction; however, it is not available in most institutions, so we believe that the cavity shave margin technique could be a valid tool to reduce the positive margin risk.

In our retrospective study, we did not report a significant difference in terms of locoregional recurrence at 3-year follow-up. In the CS group, recurrence at 3 years of follow-up was 6.1% and 12.2% in the control group. Assuming that many DCIS lesions could be indolent, recurrence could not be necessarily associated with resection margins (18, 19). As reported in our previous analysis, a positive margin in indolent DCIS may also never lead to recurrence due to the nonprogression of the tumor (20). This hypothesis could explain the absence of a difference in terms of recurrence, regardless of the high incidence of positive margins in the C group. We strongly believe, as many researchers in the scientific community do, that gene biosignature can predict recurrence risk (21–24). In patients subjected to cavity shaving, we reported a significant reduction in operative time of about 25 min compared with the control group. A different result was reported by Mohamedahmed et al. (25). In their analysis, the authors reported a longer surgical time when cavity shaving is performed (79 min ± 4 min vs. 67 min ± 3 min; mean difference: 12.14; *p* = 0.002) (25). In the analysis by Mohamedahmed et al., the control group was not subjected to intraoperative evaluation of resection margins. Differently, Monib et al. reported that cavity shaves, ensuring microscopic clearance, do not increase operating time (26). We reported a longer operative time in the C group; this is most likely linked to the technical time needed for the intraoperative evaluation of surgical margins.

The main limitations of our study include its retrospective nature, limited small sample size, and the short follow-up period. In addition, the choice of surgical technique is often led by the surgeon’s preference based on the type of the lesion and their own personal experience.

## 5 Conclusion

One of the main challenges in BCS for patients with a DCIS diagnosis is obtaining clear margins while preserving healthy tissues. This retrospective study highlights how the cavity shave margin technique results in a reduction in the positive margin rate and surgical re-excision. Moreover, this technique also reduces operative time. Based on these findings, the cavity shave technique should be considered a valid surgical approach for patients diagnosed with DCIS.

## Data availability statement

The original contributions presented in the study are included in the article/supplementary material. Further inquiries can be directed to the corresponding author.

## Ethics statement

The studies involving humans were approved by Comitato etico lazio area 2, Policlinico Tor Vergata, Viale Oxford 81, 00133, Roma. The studies were conducted in accordance with the local legislation and institutional requirements. The ethics committee/institutional review board waived the requirement of written informed consent for participation from the participants or the participants’ legal guardians/next of kin because of retrospective nature of the study.

## Author contributions

GV: Conceptualization, Methodology, Supervision, Writing – original draft, Writing – review & editing. MP: Conceptualization, Formal Analysis, Investigation, Methodology, Writing – original draft, Writing – review & editing. MM: Data curation, Formal Analysis, Writing – review & editing. VM: Data curation, Writing – review & editing. VU: Data curation, Writing – review & editing. AN: Formal Analysis, Writing – review & editing. OB: Supervision, Writing – review & editing.
